# Supplementary material for: Investigation of the Impact of Point Defects in InGaN/GaN Quantum Wells with High Dislocation Densities
Source: Nanomaterials (Basel). 2023 Sep 16;13(18):2569. doi: 10.3390/nano13182569 (PMC10537355; doi:10.3390/nano13182569)
Supplement: Supplementary file 1 [file nanomaterials-13-02569-s001.zip › nanomaterials-2603239-supplementary.pdf]

Supplementary Material: Investigation of the impact of point defects in InGaN/GaN quantum wells with high dislocation densities

Pierre Lottigier,[1] Davide Maria Di Paola,[1] Duncan T. L. Alexander,[2] Thomas F. K. Weatherley,[1] Pablo Sáenz de Santa María Modroño,[3] Danxuan Chen,[1] Gwénolé Jacopin,[3] Jean-François Carlin,[1] Raphaël Butté,[1] and Nicolas Grandjean[1]

[1]Advanced Semiconductors for Photonics and Electronics Laboratory, Institute of Physics, École Polytechnique Fédérale de Lausanne (EPFL), CH-1015 Lausanne, Switzerland

[2]Electron Spectrometry and Microscopy Laboratory, Institute of Physics, École Polytechnique Fédérale de Lausanne (EPFL), CH-1015 Lausanne, Switzerland

[3]Université Grenoble Alpes, CNRS, Grenoble INP, Institut Néel, 38000 Grenoble, France

S1. Impact of the V-pit diameter

To determine whether the V-pit diameter impacts the samples' efficiency, we grew on purpose a third sample (V) with V-pits akin to those reported for sample U. This was achieved by lowering the GaN growth temperature (750 °C) prior to the growth of the quantum well (QW) region (1). The sample structure is displayed in Fig. S1(b). This sample shares the same structural features as sample U, namely a very similar V-pit density of $2.4 \pm 0.2 \times 10^{10}$ cm^{-2} and an average V-pit diameter of 40 ± 4 nm (Fig. S1(e)). However, its relative IQE curve (Fig. S1(h)) is similar to that of sample R (i.e., with an intensity to irradiance ratio $I_{\mathrm{PL}}/\Pi_{\mathrm{exc}}$ peak near 5×10^5 W cm^{-2}). Thus, larger V-pits cannot explain the much higher efficiency of sample U over sample R. Instead, the introduction of an In-containing UL does play a decisive role in enhancing the efficiency of these samples. This is somehow surprising owing to the high density of threading dislocations ($n_{\mathrm{TD}} > 10^{10}$ cm^{-2}). This tends to indicate the prominent role played by point defects over dislocations.

S2. Temperature- and power-dependent photoluminescence measurements

Fig. S2 shows a sketch of the experimental setup used to perform power-dependent continuous wave (cw) photoluminescence (PL) measurements. Due to the Gaussian intensity distribution of the optical excitation, the photogenerated carrier density varies across the laser spot diameter focused onto the sample, Φ, which is given by:

$$\Phi = \frac{4\lambda f_{\mathrm{obj}} M^2}{\pi D}, \tag{S1}$$

where $\lambda = 375$ nm is the laser wavelength, $f_{\mathrm{obj}} = 2.0$ mm is the focal length of our near-UV 100× Mitutoyo microscope objective of numerical aperture NA = 0.5, $M^2 = 1.12$ is the beam quality factor, and $D = 1.2$ mm is the diameter of the collimated laser beam at the entrance of the microscope objective,

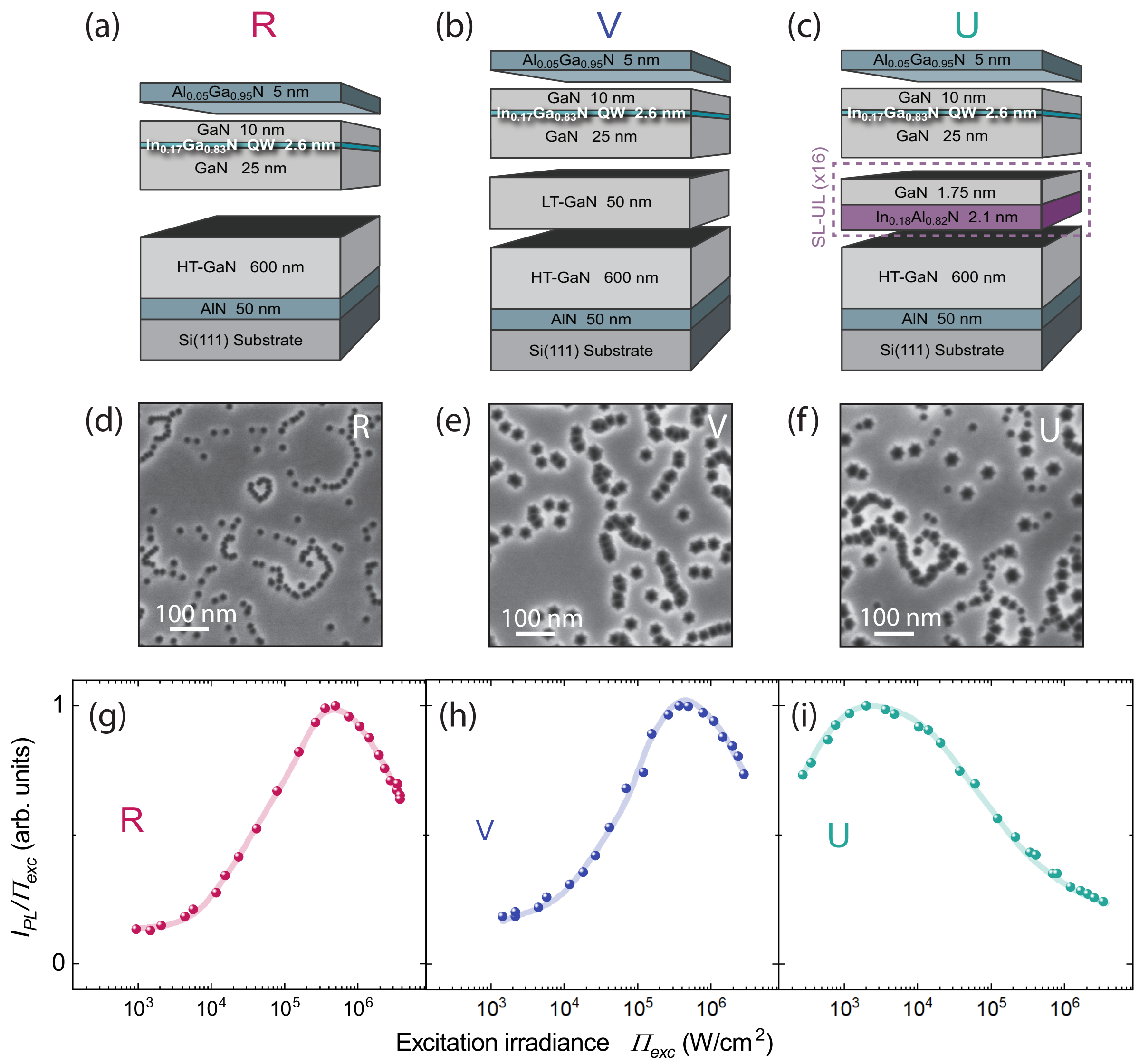

Figure S1: (a)-(c) Schematic of the samples investigated, which differ through the presence (U) or absence (R,V) of a SL-UL or the presence of a thin LT-GaN layer (V). (d)-(f) Scanning electron micrographs acquired at an accelerating voltage of 3 kV. The dark spots correspond to the V-pits that form at the termination of threading dislocations. (g)-(i) PL intensity to irradiance ratio $I_{\mathrm{PL}}/\Pi_{\mathrm{exc}}$ at room temperature, as a function of Π_{exc}. The continuous lines serve as a guide to the eye.

yielding $\Phi \approx 1\ \mu$m. The emitted luminescence is then directed toward the spectrometer. It is thus important to select only the luminescence originating from the most central part of the excitation spot, corresponding to the area with the highest and the most uniform density of photogenerated carriers. To do so, we use a spatial filtering configuration on the collection line, consisting of a lens L_1 (focal length $f_1 = 18$ cm) and of a pinhole (diameter $d = 50\ \mu$m) positioned at the focal plane of the lens. At the lens focal plane, we measured a $1/e^2$ diameter $\Phi' \sim 114\ \mu$m for the image of the PL spot, as assessed by profile measurements explained in (2). Employing a pinhole of diameter smaller than that of the imaged luminescence spot enables to filter out the light stemming from peripheral regions, characterized by lower carrier densities, and minimize any detrimental impact from in-plane carrier diffusion (3).

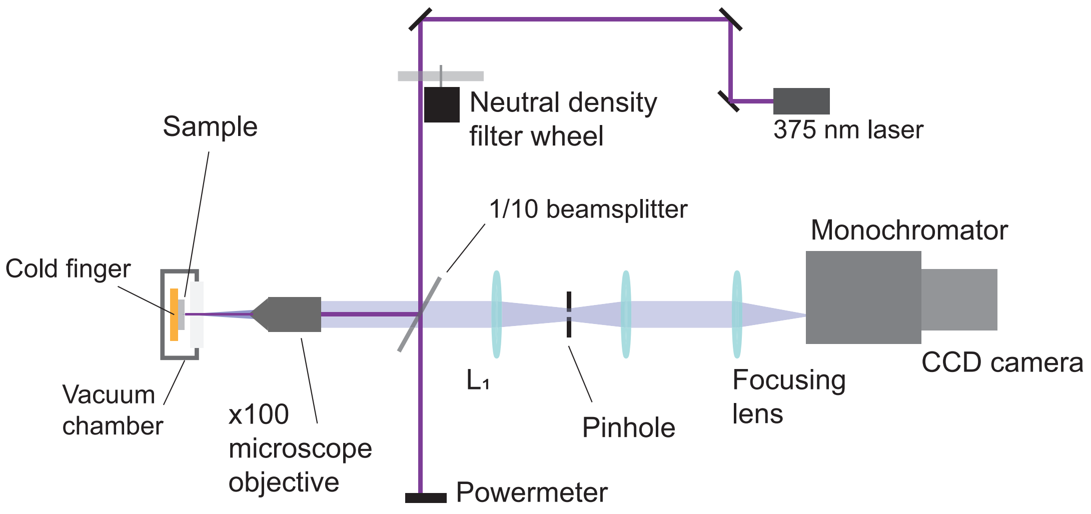

Figure S2: Schematic representation of the experimental setup employed for power-dependent cw PL measurements: a laser emitting at $\lambda = 375\,\text{nm}$ is used to excite the sample. The resulting luminescence is collected by the microscope objective used for the excitation, spatially filtered, and redirected into a spectrometer for spectral analysis.

Here, the choice of an optimal pinhole diameter is given by a trade-off between (i) selecting only the most central part of the PL spot, with the highest homogeneity in intensity, and (ii) enabling sufficient transmission of the PL signal to achieve realistic measurement conditions. Following our simulations, a pinhole diameter $d = 50\,\mu\text{m}$ was chosen in order to select only the light issued from an area illuminated with $> 70\%$ of the intensity maximum (see the intensity profile shown in Fig. S3). This also enabled us to perform PL measurements with a transmission of 30% of the integrated signal intensity through the pinhole.

S3. Modeling of cavity effects on light extraction

In the framework of this study, we employ thin films to investigate the effect of an In-containing underlayer (UL) in a dislocation-rich environment. Since the thickness of the epilayers ($L \sim 600$–800 nm) is comparable to the PL emission wavelength, it is necessary to account for cavity effects, because these may significantly alter the angular emission pattern. Indeed, microcavities embedding light emitters do not behave as Lambertian light emission sources, i.e., sources of isotropic luminance. With this aspect clearly in mind, we can first determine the cavity order, $m_c = n_{\text{eff}}L/\lambda$, as an estimate of the number of resonant modes around the emission wavelength λ in a cavity of thickness L and effective refractive index n_{eff}. In our case, $m_c \sim 4$ for the samples R, U and V, thus effectively proving that we are in the thin film limit. The single QW active medium is placed in a III-nitride multilayer structure surrounded by media that behave as partially reflecting top (air) and bottom (Si) mirrors. The light emitted from the QW layer will thus undergo multiple reflections at the top and bottom interfaces of such cavity before being extracted. Given our experimental configuration (Fig. S2), the intensity of the PL signal extracted

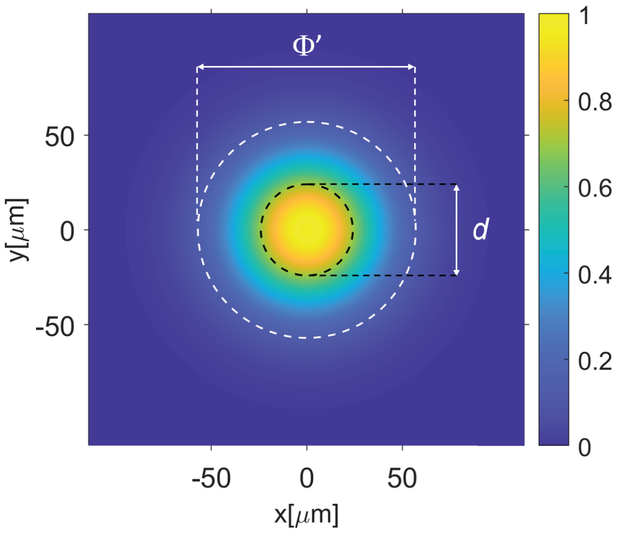

Figure S3: Normalized spatial intensity profile, I/I_0, of a PL beam with Gaussian intensity distribution, $I = I_0 \times e^{-2r^2/\Phi'^2}$, of $1/e^2$ diameter Φ' = 114 μm (white dashed curve), where $r = \sqrt{x^2+y^2}$ is the radial distance from the beam center. The part enclosed within the black dashed curve corresponds to the beam portion transmitted by the pinhole, with diameter d = 50 μm.

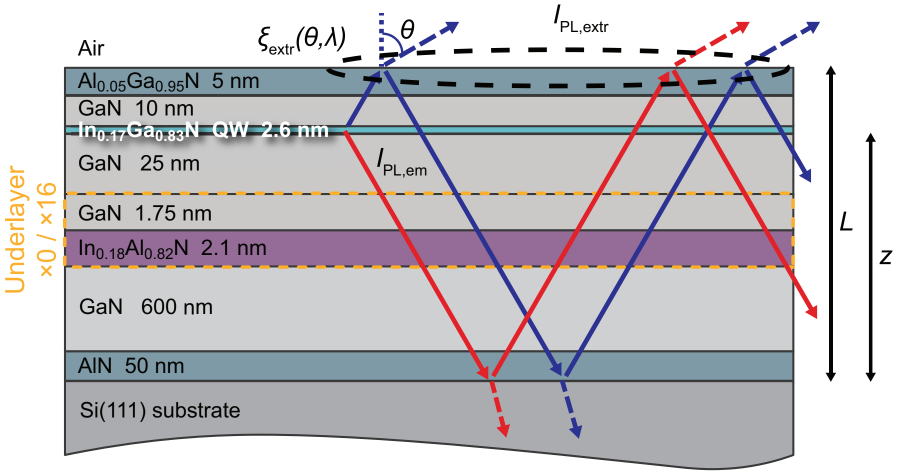

Figure S4: Sketch of the light extraction process taking place in our samples with a light source (QW layer) located at distance z from the silicon substrate embedded in a cavity of thickness L. The light extraction coefficient ($\xi_{\text{extr}}(\theta,\ \lambda)$) enables to correlate the intensity of the PL signal emitted from the source ($I_{\text{PL,em}}$) with that extracted from the cavity ($I_{\text{PL,extr}}$). 2D-FDTD calculations are based on the detailed multilayer structure considering tabulated refractive indices: AlGaN from Ref. (4), InAlN from Ref. (5), InGaN from Ref. (6) and Si from Ref. (7).

from the top surface for our samples, $I_{\text{PL,extr}}$, can be expressed as:

$$I_{\text{PL,extr}} = I_{\text{PL,em}} \times \xi_{\text{extr}}(\theta,\ \lambda), \tag{S2}$$

where $I_{\text{PL,em}}$ is the PL intensity emitted by the active region inside the cavity, and $\xi_{\text{extr}}(\theta,\ \lambda)$ is the light extraction coefficient that depends on both the PL emission external angle with respect to the normal to

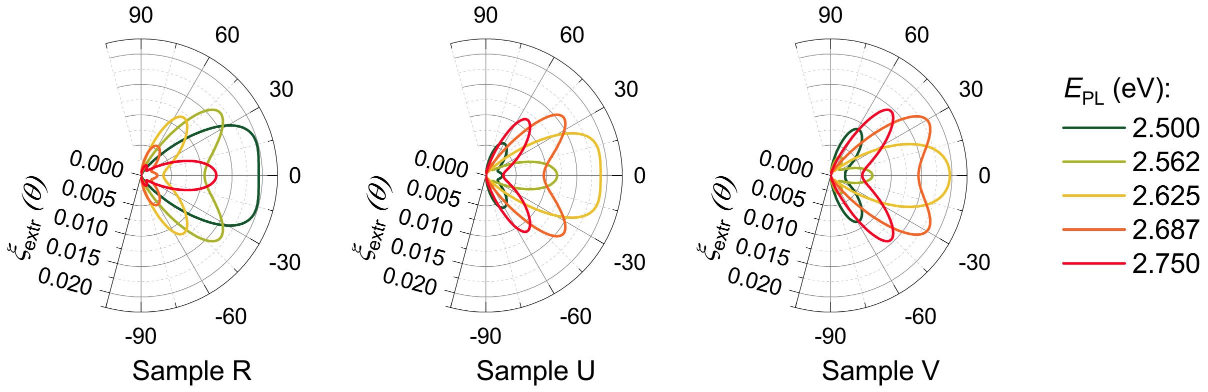

Figure S5: Light extraction coefficient ($\xi_{\text{extr}}(\theta)$), with θ given in degree, calculated at emission energies close to the QW PL emission peak for the samples of the study.

the semiconductor-air top interface (θ) and the wavelength (λ). This coefficient varies for each sample structure as it depends on the presence or not of an UL. To account for the light extraction features of these samples, the latter were modeled as a Fabry-Perot cavity of total thickness L, with an emitting QW layer placed at distance z from the silicon substrate-III-nitride epilayer interface (see sketch in Fig. S4). The emitters are set to be horizontal dipolar sources, which has been documented for compressively strained or unstrained InGaN/GaN QWs (8). The light extraction coefficient $\xi_{\text{extr}}(\theta)$ at specific emission wavelengths/energies was then obtained using a two-dimensional finite-difference time-domain (2D-FDTD) method fully compliant with our experimental geometry (9). We show in Fig. S5 the calculated values of $\xi_{\text{extr}}(\theta)$ for different values of the emission energy around the PL emission peak of the samples. These calculations show the critical dependence of $\xi_{\text{extr}}(\theta)$ upon varying angle at a given energy and upon varying wavelength/energy. Furthermore, thickness variations occurring across the wafers can also alter the light extraction coefficient. Overall, this indicates that cavity effects are at play that modify in a significant manner the intrinsic spectral lineshape of the QW PL emission for those samples. Importantly enough, the QW width (2.6 nm) yields spectral shifts due to screening of the built-in electric field upon increasing irradiance. Nonetheless, the adopted near-resonant QW excitation scheme should prevent any undesired heating effects up to the highest irradiances on the order of 10^6 W/cm^2 used in this work. As a result, due to the sole cavity effects the reconstruction of the real emitted lineshapes of the PL spectra is not possible since QW light emission is first collected through our high-NA microscope objective before spatial (real space) filtering takes place. In other words, we do not have access to the angular components (Fourier space) with our experimental configuration, hence explaining why we cannot extract and compare the absolute internal quantum efficiency (IQE) of each sample from power-dependent PL measurements. Nevertheless, as explained in the main text a reliable comparison of the efficiency of the different samples is still obtained through the dependence of the PL intensity to irradiance ratio $I_{\text{PL}}/\Pi_{\text{exc}}$ upon irradiance and the irradiance Π_{max} at which the peak efficiency is achieved.

S4. Additional temperature-dependent photoluminescence measurements evidencing local-ization

We investigate the manifestation of in-plane carrier localization in the QWs of our samples. To do so, we explore the variation of their PL emission energy with temperature, the latter being an easily accessible parameter directly influencing carrier mobility. The results are presented in Fig. S6 for samples R and U. We chose an excitation irradiance of $\Pi_{\text{exc}} = 0.25$ W cm^{-2}, low enough to be sensitive to the localization states. We see a clear S-shape for both samples knowing that such behavior has been attributed to localization phenomena in InGaN/GaN QWs (10, 11). A reported cause for localization in similar light-emitting structures is random alloy potential fluctuations (12, 13). We therefore conclude that our samples present localization that can explain the thermal activation of diffusion toward point defects for sample R: at low temperature, i.e., below $T = 50$–70 K, carriers cannot reach point defects. This explains the similar quantum efficiency of both samples at low temperature despite the presence of point defects in sample R only.

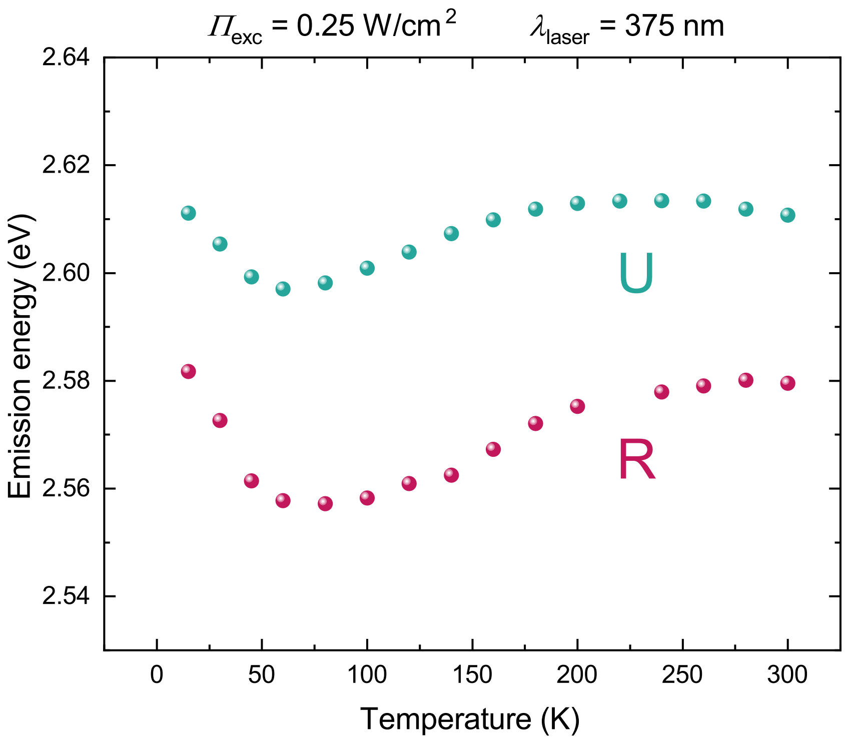

Figure S6: PL emission energy as a function of measurement temperature, acquired at an excitation irradiance as low as $\Pi_{\text{exc}} = 0.25$ W/cm^2.

S5. Time-resolved photoluminescence (TRPL)

Here we give details about the method for performing time-resolved photoluminescence (TRPL) measurements: The pulsed laser emits $\sim 1\ \mu$W pulses at a wavelength of 375 nm and at a repetition rate of

500 kHz to excite our samples. Its beam diameter of $\sim 3\ \mu$m allows to average local fluctuations inherent to the samples. The resulting luminescence is collected using a parabolic mirror and analyzed by means of a Horiba iHR550 spectrometer equipped with a 600 grooves/mm grating. With this configuration, the temporal resolution is estimated to be < 50 ps. Fig. S7 shows the TRPL decay profiles recorded at different temperatures for the two samples R and U. The effective carrier lifetimes are extracted by monoexponentially fitting the early decay regime of each curve.

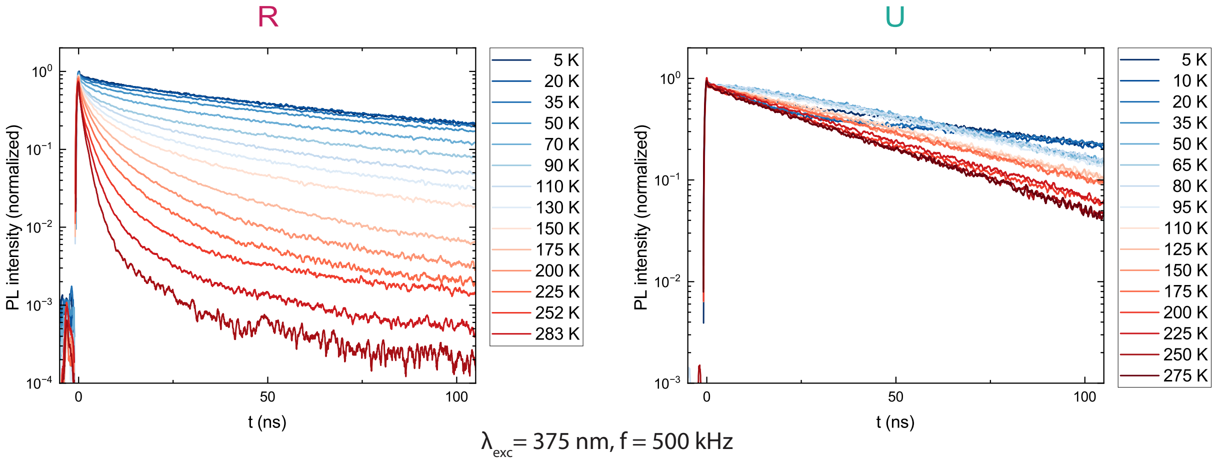

Figure S7: TRPL temporal decay profiles recorded at temperatures from $T = 5$–290 K.

S6. Cathodoluminescence measurements and diffusion

For the cathodoluminescence (CL) measurements, we used an Attolight Rosa 4364 system under an acceleration voltage of 2 kV and an average probe current in the 200–500 pA range. As explained in the main text, each CL map corresponds to a hyperspectral map for which each pixel represents the integrated intensity after the corresponding position of the sample was excited. The underlying physical process occurs as follows:

1. Energy is deposited by means of an electron beam in an excitation volume consisting mostly of GaN whose diameter ~ 9.4 nm was simulated by Monte-Carlo simulations (see Fig. S8).

2. The diffusion of the excitation beam occurs in the range of the excitation volume depth ~ 17 nm, meanwhile hot carriers relax over a mean distance of ~ 50 nm.(14) We can roughly account for all of these effects by summing their relevant scales in quadrature:

$$\sqrt{9.4^2 + 50^2 + 17^2} = 54 \text{ nm}$$

3. Provided non-radiative recombinations can be ignored in such a volume of typical dimension 54 nm, the generated carriers will quickly relax to the InGaN QW.

4. Within the QW, the carriers can diffuse in a two-dimensional landscape for some hundreds of nanometers as calculated in the main text and as reported in the literature (3).

5. In an excitation range where the Auger process is not significant, if the carriers do not encounter any non-radiative recombination center, they will recombine radiatively by emitting photons.

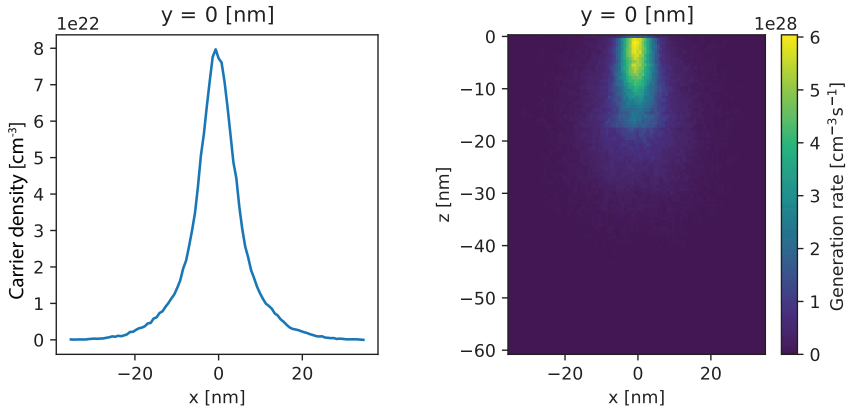

Figure S8: Monte-Carlo simulation of the volume of energy deposition by the CL electron beam.

S7. Atomic force microscopy measurements and step bunching

Fig. S9 shows atomic force microscopy (AFM) images taken on samples R and U, using super sharp tips for high resolution. On this figure, we can see areas partly encircled by V-pit arrays, associated to growth grains. The linescans within a domain display atomically flat surfaces (blue-grey lines in Figs. S9(c)-(d)) while abrupt changes in height are the signature of step-bunching. Such high misorientations are known to reduce In incorporation in the QWs (15), thereby creating energy barriers to electron-hole pairs and/or excitons. As a consequence, carriers experiencing this type of landscape will not be able to diffuse across different domains.

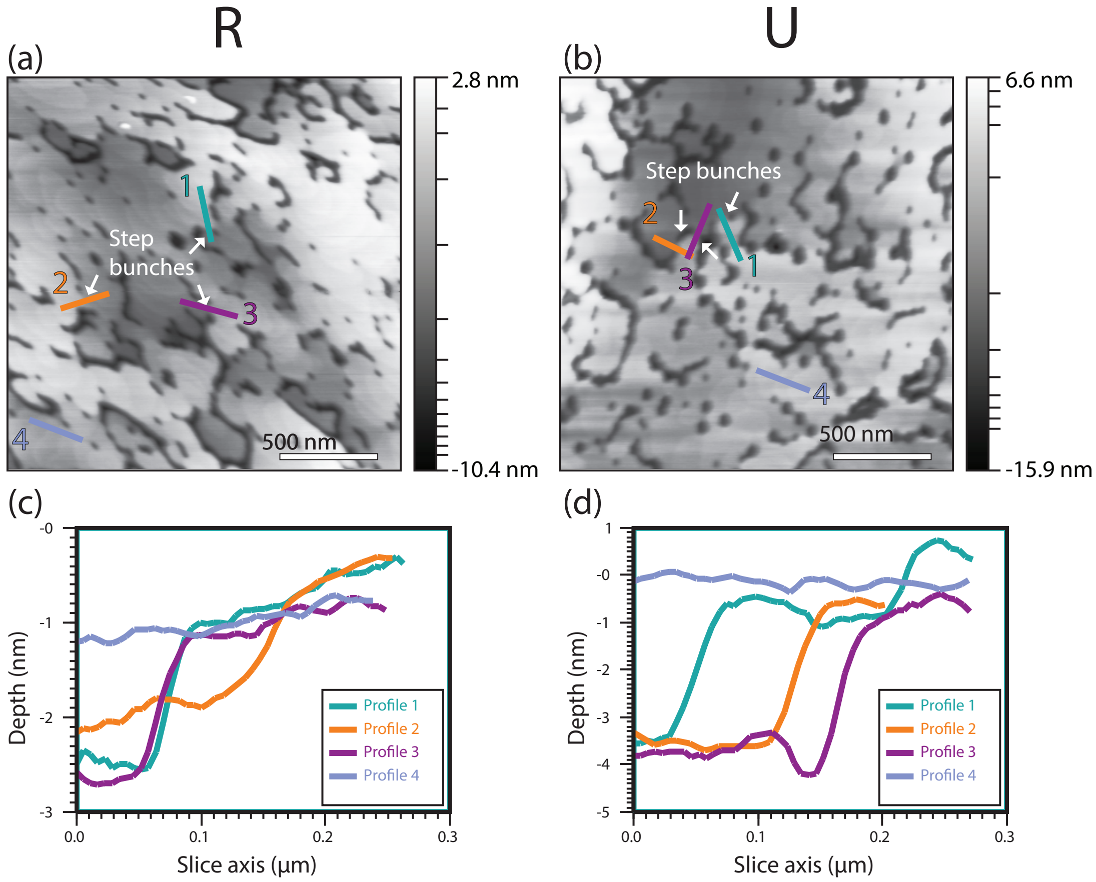

Figure S9: (a)-(b) AFM images taken on samples R and U. Arrows point toward typical domain delimitations between V-pits where step-bunching is clearly visible. (c)-(d) Height profiles extracted from the AFM maps.

References

1. S. Zhou et al., Sci. Rep. **8**, 11053 (2018).
2. K. Purvis, R. Cisek, D. Tokarz, J. Chem. Educ. **96**, 1977–1981 (2019).
3. A. David, Phys. Rev. Appl. **15**, 054015 (2021).
4. D. Brunner et al., J. Appl. Phys. **82**, 5090–5096 (1997).
5. J.-F. Carlin et al., Phys. Stat. Sol. B **242**, 2326–2344 (2005).
6. M. J. Bergmann, H. C. Casey, J. Appl. Phys. **84**, 1196–1203 (1998).
7. D. E. Aspnes, A. A. Studna, Phys. Rev. B **27**, 985–1009 (1983).
8. A. David, PhD thesis, École Polytechnique, Palaiseau, France, 2005, p. 38.
9. Department of Information Technology, Ghent University, CAMFR, version of 2012, (`http://camfr.sourceforge.net/`).
10. P. G. Eliseev, P. Perlin, J. Lee, M. Osinski, Appl. Phys. Lett. **71**, 569–571 (1997).

11. Y.-H. Cho et al., Appl. Phys. Lett. **73**, 1370–1372 (1998).

12. S. F. Chichibu et al., Nat. Mater. **5**, 810–816 (2006).

13. M. Filoche et al., Phys. Rev. B **95**, 144204 (2017).

14. U. Jahn et al., Phys. Rev. Appl. **17**, 024017 (2022).

15. M. Sarzyski et al., Appl. Phys. Express **5**, 021001 (2012).
